# Long-lasting effect of obesity on skeletal muscle transcriptome

**DOI:** 10.1186/s12864-017-3799-y

**Published:** 2017-05-25

**Authors:** Ilhem Messaoudi, Mithila Handu, Maham Rais, Suhas Sureshchandra, Byung S. Park, Suzanne S. Fei, Hollis Wright, Ashley E. White, Ruhee Jain, Judy L. Cameron, Kerri M. Winters-Stone, Oleg Varlamov

**Affiliations:** 1 School of Biological Sciences, University of California, Irvine, Irvine, CA 92697 USA; 2 Division of Cardiometabolic Health, Oregon National Primate Research Center, L584 505 NW 185th Ave., Beaverton, OR 97006 USA; 3 Division of Biomedical Sciences, School of Medicine, University of California, Riverside, Riverside, CA 92521 USA; 4 Department of Public Health and Preventive Medicine, Oregon Health and Science University, Portland, OR 97239 USA; 5 Division of Neuroscience, Oregon National Primate Research Center, Beaverton, OR 97006 USA; 6 Department of Neuroscience and Psychiatry, University of Pittsburgh, Pittsburgh, PA 15260 USA; 7 Department of School of Nursing, Oregon Health and Science University, Portland, OR 97239 USA

**Keywords:** Insulin resistance, Caloric restriction, High-fat diet, Skeletal muscle, Obesity

## Abstract

**Background:**

Reduced physical activity and increased intake of calorically-dense diets are the main risk factors for obesity, glucose intolerance, and type 2 diabetes. Chronic overnutrition and hyperglycemia can alter gene expression, contributing to long-term obesity complications. While caloric restriction can reduce obesity and glucose intolerance, it is currently unknown whether it can effectively reprogram transcriptome to a pre-obesity level. The present study addressed this question by the preliminary examination of the transcriptional dynamics in skeletal muscle after exposure to overnutrition and following caloric restriction.

**Results:**

Six male rhesus macaques of 12–13 years of age consumed a high-fat western-style diet for 6 months and then were calorically restricted for 4 months without exercise. Skeletal muscle biopsies were subjected to longitudinal gene expression analysis using next-generation whole-genome RNA sequencing. In spite of significant weight loss and normalized insulin sensitivity, the majority of WSD-induced (*n* = 457) and WSD-suppressed (*n* = 47) genes remained significantly dysregulated after caloric restriction (FDR ≤0.05). The Metacore^TM^ pathway analysis reveals that western-style diet induced the sustained activation of the transforming growth factor-β gene network, associated with extracellular matrix remodeling, and the downregulation of genes involved in muscle structure development and nutritional processes.

**Conclusions:**

Western-style diet, in the absence of exercise, induced skeletal muscle transcriptional programing, which persisted even after insulin resistance and glucose intolerance were completely reversed with caloric restriction.

**Electronic supplementary material:**

The online version of this article (doi:10.1186/s12864-017-3799-y) contains supplementary material, which is available to authorized users.

## Background

Caloric surplus brought about by a calorie-dense, high-fat Western-style diet (WSD) is an underlying risk factor for obesity, insulin resistance (IR), and type-2 diabetes [1], with hyperglycemia and dyslipidemia playing a central role in the development of diabetic complications, including β-cell dysfunction, postprandial hyperglycemia, microvascular dysfunction, and diabetic retinopathy [2–6]. Paradoxically, many obese patients who exhibit good glycemic control through lifestyle modification or medical intervention continue to experience metabolic complications [7]. This phenomenon has been termed programming, or “metabolic memory”, and it has been proposed that long-lasting metabolic complications are due to sustained epigenetic modifications [8–12].

Skeletal muscle (SM) metabolic memory has been recently defined [13], but the underlying transcriptional regulation and physiological relevance are poorly understood. For example, SM metabolic memory has been demonstrated in human studies, showing that transient exposure to high-fat diet introduced sustained DNA methylation marks that were only partially erased after diet reversal [14]. One physiological function that is likely to be particularly affected by diet-induced metabolic memory is whole-body glucose disposal. SM is estimated to be responsible for up to 70% of total glucose uptake in humans, playing a major role in etiology of metabolic disease [15]. Although acute lipid infusion results in transient IR, as evidenced by an increase in inhibitory serine phosphorylation of insulin receptor substrate-1 (IRS1) [16, 17], chronic overnutrition can induce long-term transcriptional and physiological changes in SM. For example, high-fat diet is associated with the development of a local proinflammatory response [18, 19], activation of transforming growth factor-β (TGFβ) signaling [20], and the remodeling of the extracellular matrix (ECM) in SM [21–24].

One possible approach that can help reverse metabolic memory and its physiological side effects is the use of caloric restriction (CR), which is known to improve obesity outcomes in humans [25–27] and nonhuman primates (NHPs) [28, 29], reducing cardiovascular and metabolic disease risks after 4–6 months of intervention [25, 26, 30–32]. Hence, we conducted a preliminary study aimed at identifying differentially expressed SM genes associated with the development of WSD-induced obesity, and the extent to which short-term CR reverses gene expression.

## Methods

### Animals and diets

Six male rhesus macaques (Indian origin) of 12–13 years of age were housed individually, with the cage size adjusted to animal weight according to the USDA Cage Size Guide, 8th Edition. Individual housing allowed us to mimic a sedentary lifestyle while accurately quantifying physical activity and food intake. The use of chow diet and WSD for metabolic studies in rhesus macaques has been previously described by our group and other ONPRC investigators [33–35]. Chow diet consisted of two daily meals of the Fiber-balanced Monkey Diet (15% calories from fat, 27% from protein, and 59% from carbohydrates; no. 5052; Lab Diet, St. Louis, MO). WSD diet consisted of two daily meals of the TAD Primate Diet (5LOP) (36% calories from fat, 18% from protein, 45% from carbohydrates, 5A1F, Lab Diet).

Fiber-balanced Monkey Diet contains significantly lower fraction of high-glycemic carbohydrates (sucrose, fructose and lactose) and a higher proportion of low-glycemic fibers compared to TAD Primate Diet. Fiber-balanced Monkey Diet is primarily composed of non-animal products, resembling a vegetarian diet. TAD Primate Diet is closer in its composition to a calorie-rich high-fat American diet, which contains the significant proportion of saturated animal fats, cholesterol, and high-glycemic carbohydrates.

**Fig. 1:**
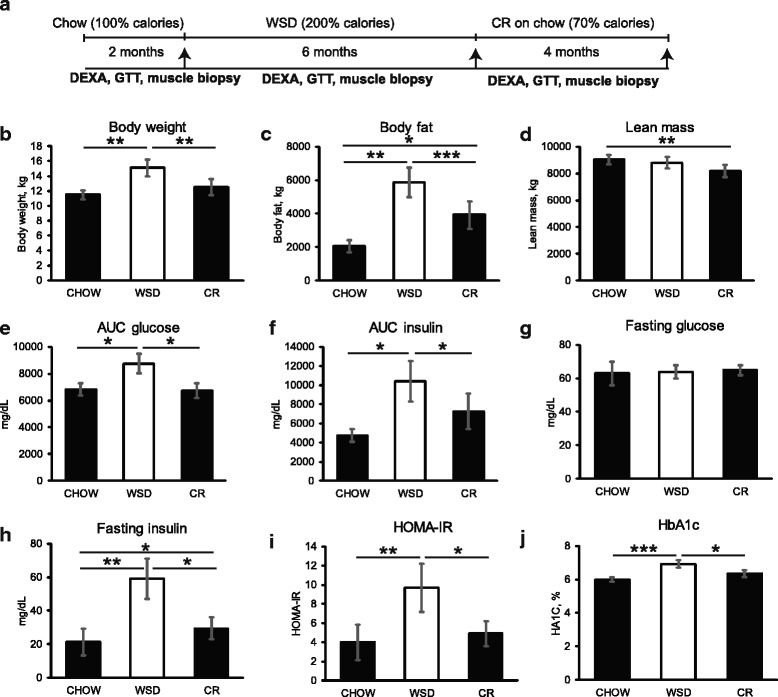
WSD-induced weight gain and insulin resistance are reversed by CR. **a** Study design. Animals were maintained in individual housing while consuming chow diet for the first 2 months, followed by Western-style diet (WSD) for 6 months, and caloric restriction (CR) on chow for 4 months. Experimental procedures (DEXA, GTT and muscle biopsies) were performed at the end of each dietary period. Body weight (**b**), total body fat (**c**), and lean mass (**d**) were determined by DEXA, and AUC glucose (**e**), AUC insulin (**f**), fasting glucose (**g**) and fasting insulin (**h**) were determined by GTT, as described in “Materials and Methods”. HOMA-IR (**i**) was calculated as described [92]. HbA1c (**j**) was determined during GTT. *Error bars* are Means ± SEM, *n* = 6. *p < 0.05, **p < 0.01 by repeated-measure one-way ANOVA

Before initiation of the study, all animals consumed *ad libitum* chow diet. After initiation of individual housing, animals were maintained for 2 months on *ad libitum* chow diet. During this period, individual baseline caloric intake was determined based on a number of consumed chow biscuits. After 2 months on chow, animals were switched to *ad libitum* WSD for 6 months (Table 1). The WSD was discontinued after 6 months because HbA1c values reached prediabetic values (Table 1). Four-month caloric restriction was performed using a chow diet, with the number of chow biscuits adjusted to 70% of individual baseline caloric intake values (Fig. 1a). During each dietary intervention, animal received similar amounts of daily fruit supplements (apple or banana).

**Table 1:** Effects of diet on physiological parameters and circulating cytokines

Parameters	Before WSD (Chow)	After WSD	After CR	WSD vs Chow	WSD vs CR	Chow vs CR
Appetite and activity						
Caloric intake, % of chow	99.7 ± 6.4	215.4 ± 19.1	ND	*p* < 0.01	ND	ND
Activity (count/day)	28624.7 ± 6842.7	33545.2 ± 5650.0	26284.0 ± 6794.2	NS	NS	NS
Body composition						
Weight (kg)	11.5 ± 0.6	15.1 ± 1.1	12.5 ± 1.1	*p* < 0.01	*p* < 0.01	NS
Body fat (g)	2040.3 ± 357.0	5855.4 ± 881.0	3934.2 ± 820.7	*p* < 0.01	*p* < 0.001	*p* < 0.05
Lean mass (g)	9028.2 ± 354.9	8793.9 ± 437.7	8179.4 ± 443.6	NS	NS	*p* < 0.01
Glucose homeostasis						
AUC glucose (mg/dl)	6823.1 ± 440.0	8751.5 ± 718.5	6735.3 ± 538.5	*p* < 0.05	*p* < 0.05	NS
AUC insulin (mg/dl)	4750 ± 647.2	10404 ± 2078.9	7273.6 ± 1869.3	*p* < 0.05	*p* < 0.05	NS
Fasting glucose (mg/dl)	63.5 ± 7.2	63.6 ± 4.0	65.5 ± 2.8	NS	NS	NS
Fasting insulin (mg/dl)	21.5 ± 7.8	58.5 ± 12.2	29.5 ± 6.6	*p* < 0.01	*p* < 0.05	*p* < 0.05
HbA1c, percent	6.02 ± 0.15	6.92 ± 0.23	6.35 ± 0.19	*p* < 0.001	*p* < 0.05	NS
HOMA-IR	4.05 ± 1.86	9.7 ± 2.55	4.9 ± 1.3	*p* < 0.01	*p* < 0.05	NS
Serum cytokines						
IL6, % of chow	100 ± 0.0	68.9 ± 10.0	120.2 ± 7.7	NS	*p* < 0.01	NS
IL-8, % of chow	100 ± 0.0	73.5 ± 13.2	59.6 ± 11.6	*p* < 0.001	NS	*p* < 0.001
Eotaxin, % of chow	100 ± 0.0	60.7 ± 6.9	49.5 ± 3.6	*p* < 0.001	NS	*p* < 0.001
MIF Analyte, % of chow	100 ± 0.0	212.8 ± 64.4	472.9 ± 110.2	NS	*p* < 0.05	*p* < 0.01
PAI-1, ng/ml	104.2 ± 5.08	121.2 ± 4.73	125.2 ± 5.33	*p* < 0.05	NS	*p* < 0.01

Following 2 months on *ad libitum* chow diet, animals were provided with *ad libitum* access to a WSD for 6 months, and then switched back to chow, while being calorically restricted to 70% of individual baseline values. Body composition and glucose homeostasis were assessed before WSD (Chow), 6 months after WSD, and 4 months after CR. Serum cytokine and PAI-1 levels were determined before WSD, 4 months after WSD, and 4 months after CR. Daily caloric intake was determined during chow and WSD periods. Daily caloric intake and serum cytokine levels are presented as normalized a percent of baseline values. Values are Means ± SEM, *n* = 6. Statistical significance was determined using repeated-measure one-way ANOVA. *ND* not determined, *NS* not significant

### Activity monitoring

Activity was measured continuously throughout the experiment using Actical omnidirectional accelerometers (Respironics, Phoenix, AZ). Each monkey was fitted with a loose-fitting metal collar (Primate Products, Inc. Immokalee, FL) that housed the accelerometer in a snug, protective stainless steel box, as previously described [36]. Monitors were programmed to record the total number of activity counts per minute. Activity data were downloaded at least every 45 days while animals were under sedation. Total daily activity level was averaged for a 2-month baseline period, over the last week of the 6-month WSD period and over the last week of the 4-month CR period.

### Dual-energy X-ray absorptiometry

Percent body fat was determined using dual-energy X-ray absorptiometry (DEXA) scanning as described [37]. Monkeys were sedated with ketamine and positioned supine on the bed of a Hologic DEXA scanner (Discovery scanner, Hologic Inc, Bedford, MA).

### Glucose tolerance test

Each animal was sedated initially with Telazol (Tiletamine hydrochloride and Zolazepam hydrochloride, Fort Dodge Animal Health, Fort Dodge, IA) and subsequently with ketamine to maintain sedation. The protocol was based on that designed by Bergman et al. [38]. Dextrose (300 mg/kg) was infused intravenously through a catheter and blood samples were taken from 15 min before to three hours after the glucose infusion. Tolbutamide (5 mg/kg) was infused intravenously 20 min after the dextrose in order to stimulate the pancreas to secrete more insulin. All samples were immediately assayed for glucose using a YSI 2300 Stat Plus (YSI Inc., Yellow Springs, OH), and subsequently for insulin by RIA (Linco Human Insulin RIA, Millipore Corporation, Billerica, MA). Glucose and insulin were sampled at 1, 3, 5, 10, 20, 40 and 60 min after baseline. The sensitivity of the insulin assay was 1 μU/ml and the intra-assay coefficient of variation was 4.9%.

### Cytokine, chemokine, and growth factor analysis

Plasma samples from the following time points: before WSD, 4 months on a WSD, and 4 months on caloric restriction, (stored at −80 °C) were thawed and analyzed in duplicates using the Invitrogen Cytokine Monkey Magnetic 29-Plex Panel per the manufacturer’s instructions, using the Magpix spectrophotometer (Life Technologies, Grand Island, NY). The panel includes monocyte chemoattractant protein 1 (MCP-1; CCL2), fibroblast growth factor basic (FGF-β), IL-1β, granulocyte colony-stimulating factor (G-CSF), IL-10, IL-6, IL-12, RANTES, eotaxin, IL-17, macrophage inflammatory protein 1 alpha (MIP-1α), granulocyte-macrophage colony-stimulating factor (GM-CSF), macrophage inflammatory protein 1 beta (MIP-1β), IL-15, epidermal growth factor (EGF), IL-5, hepatocyte growth factor (HGF), vascular endothelial growth factor (VEGF), IFN-γ, monocyte-derived chemokine (MDC; CCL22), interferon-inducible T cell alpha chemoattractant (ITAC; CXCL11), migration inhibition factor (MIF), IL-1 receptor agonist (IL-1RA), TNF-α, IL-2, IFN-gamma-inducible protein 10 (IP-10, CXCL10) monokine induced by IFN-gamma (MIG; CXCL9), IL-4, and IL-8 (see Additional file 1: Figure S1 for details).

### Muscle biopsies

Soleus muscle are primarily comprised of slow twitch fibers [39] and has a greater sensitivity to insulin compared to fast twitch muscles [40]. Soleus muscle displays a well-documented response to obesity, as demonstrated in human [41, 42] and rhesus macaque [43] studies, representing an appropriate translational model for studying the effects of diet on SM physiology. SM biopsies were performed by expert surgical personnel at ONPRC according to well-accepted veterinary surgical procedures under sterile conditions and appropriate anesthesia with postoperative pain control. Food was withheld for approximately 12 h prior to the procedure. Animals were sedated with 100 mg ketamine combined with 0.1 mg Glycopyrrolate administered intramuscularly. Once the intravenous catheter was placed, animals received 0.5 mg Hydromorphone-HCl intravenously. Animals were endotracheally intubated with an endotracheal tube (size 4.0–6.0) and general anesthesia was induced with 3% Isoflurane for 2–3 min. Inhalant anesthesia was maintained at 1–2% Isoflurane. Inhalant anesthetics was combined with 100% oxygen administered at a rate of 1–1.5 L/min.

A 2-cm incision was made lateral to the soleus muscle. Using sharp dissection, an approximately 1 × 0.5 cm muscle specimen from the lateral aspect of soleus muscle was obtained, rinsed in saline, and snap-frozen in liquid nitrogen. This surgical procedure produced minimal amount of bleeding at the site of biopsy. Closure of the muscle fascia with simple continuous 4-0 Monocryl was followed by continuous intradermal 4-0 Monocryl in the skin. Recovery was on the OR table until extubation. Additional heat and oxygen support was provided as needed during the recovery period. Post-operative analgesia was provided for 48–72 h following the surgical procedure, using Hydromorphone HCl (0.05–0.4 mg/kg, administered intramuscularly, three times a day), and buprenorphine (0.01–0.1 mg/kg, administered intramuscularly, once a day). The standard 48 to 72-h opioid protocol for post-operative analgesia was used. Post-operative monitoring and assessment of pain and distress were accomplished by surgical veterinary staff for a minimum of 7 days.

### RNA isolation

Frozen 100-mg muscle specimens were homogenized by shaking (25 rps for 2 min) in 2-ml extraction tubes supplied with a 5-mm pre-chilled metal bead (McMaster-Carr, Elmhurst, IL) and 1 ml of ice-cold TriReagent (MRC Inc., Cincinnati, OH), using a TissueLyser II (Qiagen, Hilden, Germany). One hundred microliters bromochloropropane (MRC Inc., Cincinnati, OH) were added to each tissue sample, to enhance phase separation, and incubated at room temperature for 5 min. Samples were mixed and centrifuged at 12,000 × *g* for 15 min at 4 °C. The RNA-containing upper aqueous phase was transferred to a new tube and mixed with 12 μl glycogen (Thermo Fisher Scientific, Waltham, MA) and 0.5 ml isopropanol. Tubes were centrifuged at 15,000 × *g* for 10 min at 25 °C, pellets were washed twice with 0.7 ml 75 and 100% ethanol, air-dried at room temperature for 10–15 min, and resuspended in 10 mM Tris-HCl, pH 8.0. RNA concentration and purity were determined using NanoDrop ND1000 (Thermo Fisher Scientific). The average A260/280 value of purified RNA samples was 2.04.

### RNAseq analysis

RNAseq libraries (3 longitudinal time points/3 animals) were prepared using the TruSeq protocol (Illumina, San Diego, CA). Briefly, poly (A) + RNA was purified using oligo-dT coated magnetic beads, chemically fragmented followed by cDNA generation using random hexamer primers. The cDNAs ends were repaired and ligated to library adaptors. Following clean-up with AMPure XP beads (Beckman Coulter Inc., Brea, CA), the libraries were amplified using 11 PCR cycles. The amplified libraries were cleaned using AMPure XP beads. The library was profiled on a Bioanalyzer (Agilent, Santa Clara, CA) and quantified using qRT-PCR (Kapa Biosystems, Wilmington, MA) on a StepOnePlus qRT-PCR workstation (Life Technologies, Carlsbad, CA). Libraries were mixed for multiplexing and the final concentration of the mix was determined by qRT-PCR. The mix was diluted to 1 nM for denaturation and then diluted to deliver optimal clustering on the flow cell. Flow cells were prepared on a cBot (Illumina, San Diego, CA). Libraries were sequenced on a HiSeq 2000 (Illumina, San Diego, CA). Data was assembled into standard fastq files using Bcl2Fastq (Illumina, San Diego, CA).

### Bioinformatic analysis

Bioinformatic analysis was carried out as described [44]. The quality of the raw reads was verified using FastQC (version 0.11.3). Low quality bases as well as any remaining Illumina adapters were trimmed. Reads with less than 25 bases remaining were discarded. The remaining reads were aligned to the rhesus macaque genome (*Macaca mulatta* 1.0) from ENSEMBL using splice aware short read aligner suite Bowtie2/TopHat2 (REF1) in a strand-specific fashion allowing up to 5% mismatches. The transcript counts per gene were calculated using *SummarizeOverlaps* function (Union method) in GenomicRanges (REF2) package in R. Transcripts were normalized using trimmed mean of *M*-values (TMM) method followed by differential gene expression analysis using edgeR (REF3) resulting in candidate differentially expressed genes (DEGs), with fold change (FC) ≥ 2 and a false discovery rate (FDR) ≤ 0.05. Functional enrichment was done using MetaCore (GeneGo™, Thomson Reuters, NY).

### Quantitative RT-PCR (qRT-PCR) analysis of gene expression

Two micrograms of mRNA samples were used for cDNA synthesis using the SuperScript VILO^TM^ cDNA Synthesis kit (Thermo Fisher Scientific). cDNA was diluted 1:10 and 1 μl was used in 10 μl of qRT-PCR reactions using the Power SYBR Green Master mix (Thermo Fisher Scientific), according to manufacturer’s instructions. qRT-PCR was performed using an ABI7900 thermocycler (Applied Biosystems, Inc). Primer sequences are shown in Additional file 2: Table S4. The data were normalized to the RPL13A housekeeping gene and the fold change was calculated using 2^-ddCT method.

### Statistical analysis

All data was checked for normality and homogeneity of variance. If necessary, data was transformed using log or square root transformations to meet criteria for parametric tests. Comparisons between the baseline time period and WSD and CR periods were made using repeated measure one-way ANOVA, with a Bonferroni correction for multiple comparisons. Data are presented as mean ± standard error of the mean (SEM). All statistical analyses were conducted using the SPSS software package, version 23.0 (SPSS Inc., Chicago, Illinois).

## Results

### Diet effects on physiological parameters

Over the course of the WSD, the total caloric intake increased significantly compared to the chow period. In contrast, total physical activity levels were not significantly affected either by a WSD or CR (Table 1 and Fig. 1a). Body weight and fat mass increased significantly following consumption of the WSD. Although fat mass decreased significantly following CR, it did not return to baseline levels. In contrast, lean mass was not affected by the WSD, but decreased significantly after CR compared to the chow period (Table 1 and Fig. 1b–d).

WSD induced glucose intolerance and IR as evidenced by an increase in area under the curve (AUC) glucose and AUC insulin values during glucose tolerance tests (GTTs), as well as HOMA-IR and HbA1c levels (Table 1 and Fig. 1e, f, i and j). CR significantly decreased these parameters, suggesting a return to normal glucose tolerance and improved insulin sensitivity following weight loss. Fasting insulin, but not fasting glucose, increased significantly following WSD, and then decreased after CR, albeit remaining significantly elevated compared to the chow period (Table 1 and Fig. 1g and h). Collectively, CR restored normal glucose tolerance and insulin sensitivity to its normal level while having only a partial effect on fat loss in rhesus macaques exposed to a WSD.

### Diet effects on circulating cytokines

Circulating levels of interleukin-6 (IL-6) were not significantly affected by WSD, but increased significantly after CR. Circulating IL-8 levels were decreased after WSD and remained significantly reduced after short-term CR. Similarly, serum concentrations of eotaxin (CCL11) decreased significantly after a WSD and remained low during CR. In contrast, serum concentrations of macrophage migration inhibitory factor (MIF) almost doubled after WSD and then quadrupled after short-term CR (Table 1). Circulating levels of plasminogen activator inhibitor 1 (PAI-1) were elevated by WSD and remained elevated after CR (Table 1). The Luminex analysis of 29 circulating cytokines, chemokines and growth factors is shown in Additional file 1: Figure S1.

### Diet effects on skeletal muscle gene expression

#### WSD induces sustained transcriptional changes that largely persist after CR

**Fig. 2:**
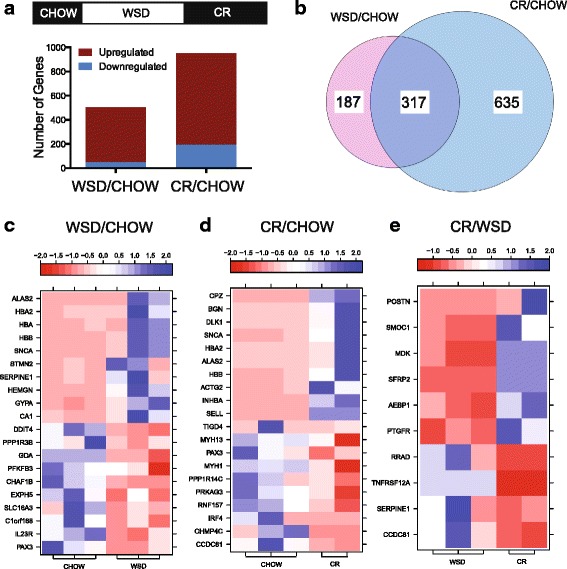
WSD induces sustained alterations in skeletal muscle transcription. Soleus muscle biopsies were collected longitudinally, before and after exposure to the WSD and after CR, and then subjected to RNAseq gene expression analysis, as described in “Materials and Methods”. DEGs were identified using three independent comparisons: **a**–**c** WSD vs chow (*WSD/CHOW*); **e** CR vs WSD (*CR/WSD*); and **a**, **b** and **d** CR vs chow (*CR/CHOW*). **a** The number of upregulated (*brown*) and downregulated (*blue*) genes from *WSD/CHOW* and *CR/CHOW* categories in rhesus macaque genome. **b** Venn diagram shows an overlap between *WSD/CHOW* and *CR/CHOW* genes. Heat maps of top DEGs during the transition from chow to WSD (**c**), CR vs chow (**d**), and CR vs. WSD (**e**) Each column represents an individual animal

To study diet-induced changes in the SM transcriptome, soleus muscle biopsies were collected longitudinally, before and after exposure to the WSD and following CR, and then subjected to RNAseq gene expression analysis. Differentially expressed genes (DEGs) were identified using three comparisons: *WSD/CHOW*, *CR/WSD*, and *CR/CHOW* (Fig. 2). This experimental design allowed us to identify DEGs whose expression reversed or remained resistant to CR. More than 90% of *WSD/CHOW* DEGs were upregulated (total gene count: DEG_up_ = 457; DEG_down_ = 47; Additional file 2: Table S1 and Fig. 2a). Similarly, 80% of *CR/CHOW* DEGs were also upregulated (total gene count: DEG_up_ = 761, DEG_down_ = 191; Additional file 2: Table S2 and Fig. 2a). Many DEGs identified in the present study are found to be expressed in human erythroid cells [45] (erythroid gene count: *WSD/CHOW* DEG_up_ = 244, DEG_down_ = 15; *CR/CHOW* DEG_up_ = 375, DEG_down_ = 67; Additional file 2: Table S3). This analysis suggests that red blood cells residing in the intramuscular capillary system may contribute to SM gene expression, or alternatively, some of these genes are expressed both in SM and erythroid cells.

#### Only a small subset WSD-affected DEGs shows reversible regulation by CR

There were 317 genes whose expression was significantly affected by a WSD and remained dysregulated after CR (Fig. 2b). Moreover, only 10 *CR/WSD* DEGs were identified in the present study (Fig. 2e and Table 2), suggesting that only few of the WSD-induced and WSD-suppressed genes are reversed by CR. Some of the notable genes in the CR/WSD list included *SERPINE1*, whose expression was initially upregulated by a WSD, and then reversed by CR. Other reversed genes included Ras-related associated with diabetes (*RRAD*) and the tumor necrosis factor receptor superfamily-12A (*TNFRS12A)* (Fig. 2e and Table 2).

**Table 2:** The effect of CR on WSD-induced gene expression

Gene symbol	Description	Log_FC	*P*-Value	FDR
POSTN	Periostin, osteoblast specific factor	3.18	1.46E-06	0.0025
SMOC1	SPARC related modular calcium binding 1	1.97	1.30E-07	0.0005
MDK	Midkine (neurite growth-promoting factor 2)	1.82	1.11E-05	0.02
SFRP2	Secreted frizzled-related protein 2	1.48	2.80E-07	0.0006
AEBP1	AE binding protein 1	1.41	1.00E-06	0.002
PTGFR	Prostaglandin F receptor	1.31	2.72E-05	0.03
RRAD	Ras-related associated with diabetes	−1.57	1.61E-07	0.0005
TNFRSF12A	Tumor necrosis factor receptor superfamily 12A	−1.71	8.20E-08	0.0005
SERPINE1	Serpin peptidase inhibitor, clade E	−2.53	2.20E-05	0.03
CCDC81	Soiled-coil domain containing 81	−2.68	2.43E-08	0.0003

Soleus muscle biopsies were collected longitudinally, before WSD, 6 months after WSD, and 4 months after CR. Tissue samples were subjected to RNA extraction and RNAseq gene expression and bioinformatic analysis as described in “Materials and Methods”. Table shows upregulated and downregulated differentially expressed genes (DEGs) after CR in comparison with WSD (CR/WSD). Bioinformatic analysis was performed using FDR ≤ 0.05. Log_FC (fold change) was calculated as Log (FC, 2)

#### The most upregulated and most downregulated genes affected by diet

Genes that were highly upregulated by the WSD encoded the heme biosynthesis enzyme 5′-aminolevulinate synthase 2 (*ALAS2*), several isoforms of hemoglobin (*HBA2, HBA, HBB*), the regulator of hematopoietic cell proliferation hemogen (*HEMGN*), and the erythrocyte sialoglycoprotein glycophorin A (*GYPA*) (Fig. 2c and Additional file 2: Table S1). WSD induced the downregulation of several metabolic genes, encoding glycolytic enzymes 6-phosphofructo-2-kinase/fructose-2,6-bisphosphatase-3 and 1 (*PFKFB3* and *1*), the glycogen synthesis regulatory gene, protein phosphatase 1, regulatory subunit 3B (*PPP1R3B*), uncoupling protein 3 (*UCP3*) and the negative regulator of mTORC, DNA-damage-inducible transcript 4 (*DDIT4*) (Fig. 2c and Additional file 2: Table S1). The most upregulated genes in the *CR/CHOW* comparison were related to ECM remodeling, including metallopeptidase carboxypeptidase Z (*CPZ*), biglycan (*BGN*), selectin L (*SELL*) and several types of collagen. The most downregulated genes in the *CR/CHOW* group included tiger transposable element derived 4 (*TIGD4*), the developmental transcription factor *PAX3*, myosin heavy chain 1 and 13 (*MYH1* and *MYH13*), and interferon regulatory factor 4 (*IRF4*, Fig. 2d and Additional file 2: Table S2).

#### TGFβ pathway and adhesion molecule genes remain upregulated after CR

**Fig. 3:**
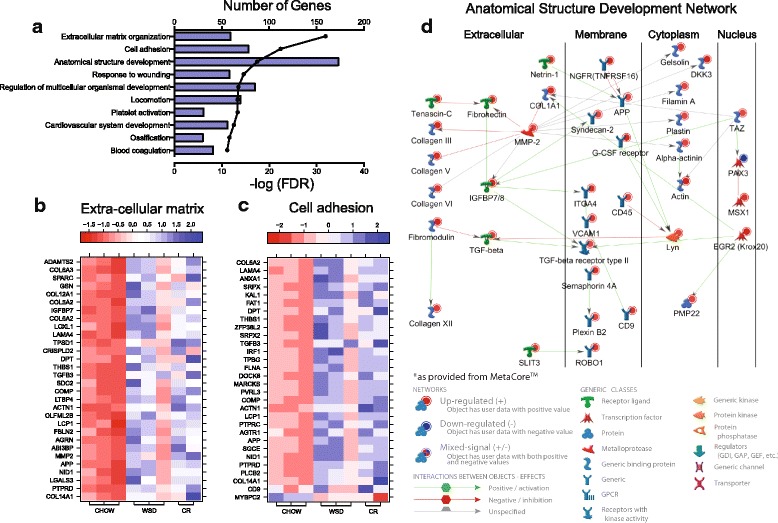
TGFβ pathway genes remain upregulated after CR. **a** Functional enrichment of DEGs with the highest FDR values common for *WSD/CHOW* and *CR/CHOW* categories. Heat maps of top DEGs involved in ECM organization (**b**) and cell adhesion (**c**). Each column represents an individual animal. **d** Anatomical structure development common gene network indicates upregulated (*red*) and downregulated (*blue*) DEGs. Positive and negative interactions between genes are represented by *green* and *red arrows*, respectively. Cellular compartmentalization of gene products is indicated

We identified 317 genes whose expression was significantly affected by a WSD but not reversed by CR (Fig. 2b). Functional enrichment was performed using Metacore™ and showed that these DEGs enriched to gene ontology (GO) terms associated with ECM organization and cell adhesion (Fig. 3a–c). The former included multiple isoforms of collagens, such as *COL4A1, COL5A2, COL6A2, COL6A3, COL12A1, and COL14A1*, the ECM remodeling metalloproteases *ADAMTS2,* and matrix metalloprotease *MMP2* (Fig. 3b). Adhesion molecule genes included laminin *LAMA4* gene*,* cadherin-like gene *FAT1*, and thrombosponin-5 gene *COMP* (Fig. 3c). The network analysis of DEGs that map to the GO term “anatomical structure development” and are known to directly interact show that genes encoding several members of the TGFβ − signaling pathway remains upregulated after the diet switch to CR (Fig. 3d).

#### Muscle development genes affected by caloric restriction

**Fig. 4:**
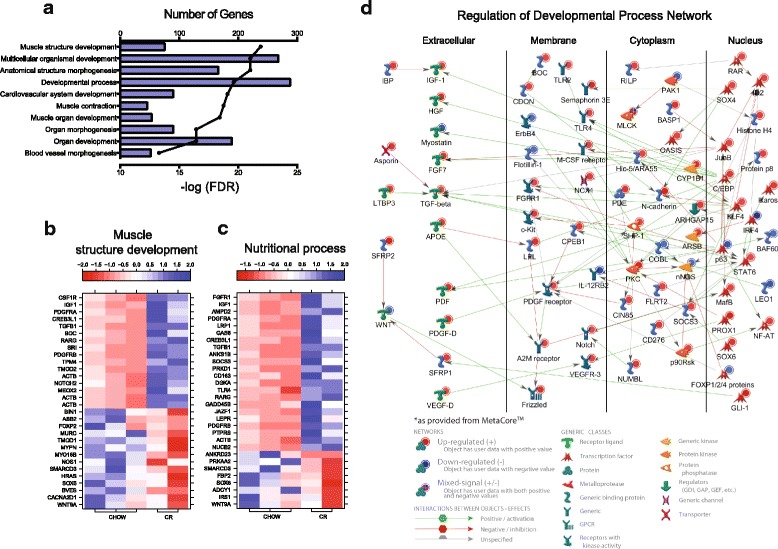
CR-specific gene regulation. **a** Functional enrichment of *CR/CHOW*-specific DEGs with the highest FDR values that *excludes* DEGs present in *WSD/CHOW* and CR/WSD categories. Heat maps of top CR-specific DEGs involved in muscle structural development (**b**) and nutritional processes (**c**). Each column represents an individual animal. **d** CR-specific regulation and development gene network indicates upregulated (*red*) and downregulated (*blue*) DEGs. Positive and negative interactions between genes are represented by *green* and *red arrows*, respectively. Cellular compartmentalization of gene products is indicated

We detected 635 differentially expressed *CR/CHOW* genes that were enriched to GO terms associated with muscle development (Fig. 4a). Among the downregulated muscle development genes were the wingless-type MMTV integration site family, member 9A (*WNT9A*) involved in the neuromuscular junction formation [46], the transcription factor *SOX6* whose deficiency is associated with an increased number of slow myofibers and a decreased number of fast myofibers [47], and nitric oxide synthase (*NOS1*), a positive regulator of SM hypertrophy [48] (Fig. 4b). Several other downregulated genes important for muscle development include BIN1/M-Amphiphysin2 (*BIN1*), which is frequently mutated in centronuclear myopathies [49], and muscle-restricted coiled-coil (*MURC*) playing the pivotal role in skeletal myogenic differentiation [50] (Fig. 4b–d). Thus, downregulation of these genes may collectively account for muscle loss after CR (Fig. 1d and Table 1).

#### Energy metabolism and other genes affected by caloric restriction

**Fig. 5:**
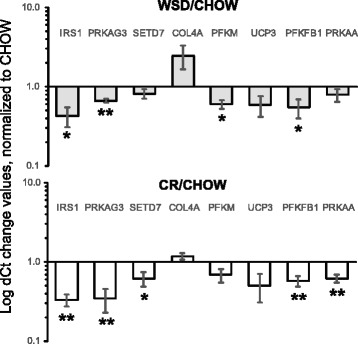
qRT-PCR validation of RNAseq analysis of gene expression. Changes in SM mRNA levels were determined as described in “Materials and Methods”. Graphs represent logarithm mean fold changes for WSD Ct values normalized to CHOW (*filled bars*) and CR Ct values normalized to CHOW (*open bars*). *Error bars* are Means ± SEM, *n* = 5. *p < 0.05, **p < 0.01 by repeated-measure one-way ANOVA

Additional analysis using disease terms revealed enrichment to nutritional processes (Fig. 4c). Some of the downregulated DEGs that mapped to these disease pathways include *IRS1*, fructose-1,6-bisphosphatase 2 (*FBP2*) and *PRKAA2* genes (Fig. 4c). Other disease-related genes that were upregulated by CR were the diabetes-related ankyrin repeat protein (*ANKRD23*), whose expression in SM is increased under diabetic conditions [51] and the growth arrest-specific 6 (*GAS6*) gene, which is strongly associated with adiposity, inflammation, and insulin resistance status among overweight people [52] (Fig. 4c). Interestingly, the expression of several inflammatory genes such as Toll-like receptor 4 (*TLR4*), *CD163*, and *TGFB1* increased following CR (Fig. 4c and d). A network of DEGs that mapped to the GO term “developmental process” (Fig. 4d) is consistent with the sustained activation of the TGF-β network after CR. The possible mechanism of its activation may involve the positive action of the cytokine-induced STAT6 transcriptional factor, which was also upregulated during CR, and the suppressor of cytokine signaling 3 (SOCS3), which controls macrophage polarization (Fig. 4d). Using qRT-PCR, we confirmed that energy metabolism-related genes, including *IRS1*, *PRKAG3*, *PFKM, UCP3*, *PFKFB1* and *PRKAA* were significantly downregulated or showed a trend toward downregulation by WSD and CR (Fig. 5). In contrast, the gene of collagen-4a (*COL4A*) showed the opposite regulation by WSD and CR. Interestingly, the SET domain-containing lysine methyltransferase 7 (*SETD7*) was significantly downregulated after CR (Fig. 5).

## Discussion

### Transcriptional remodeling of ECM

The present study demonstrates that WSD induced SM transcriptional reprograming that remained persistent even after obesity and glucose intolerance were reversed by CR. Remarkably, 457 WSD-induced and 47 WSD-suppressed genes were not readily reversed by CR and remained upregulated or downregulated, respectively. Upregulated genes detected by our RNAseq analysis encoded ECM-related proteins, including collagens (*COL1A1, COL3A1, COL4A1, COL5A1, COL5A2, COL6A1, COL6A2, COL6A3, COL8A2, COL11A1, COL12A1, COL14A1 and COL21A1*), integrins (*ITGBL1, ITGA4* and *ITGA5*), and matrix metalloproteases (*MMP2* and *MMP25*; Additional file 2: Table S1). This finding is consistent with recent human studies, demonstrating the upregulation of *COL1*, *COL3* and *MMP2* in response to overfeeding [53]. It has been suggested that ECM remodeling is associated with the development of diet-induced IR, contributing to the pathophysiology of type 2 diabetes [24]. The importance of ECM-integrin interactions in the development of IR has been demonstrated in mice lacking integrin-alpha_2_ beta_1_ (itga2 (-/-)), as evidenced by the fact that high-fat feeding induced *COL3* and *COL4* gene expression in SM of transgenic mice, while insulin sensitivity was increased [23]. Consequent studies revealed that the deletion of MMP9, the primary enzyme that mediates the degradation of COL4 prevented the development of diet-induced IR [54].

### Relevance to metabolic memory

Because a subset of ECM genes remained dysregulated following CR, we suspected the involvement of epigenetic modifiers in transcriptional regulation. One such modifier *SETD7* was significantly downregulated following CR. SETD7 has been previously implicated in hyperglycemia-induced epigenetic activation of profibrotic genes related to nephropathies and vascular complications (*TGFβ, SERPINE1, COL1A,* and *MCP-1*), being involved in metabolic memory [9]. Metabolic memory in humans has been documented in the Diabetes Control and Complications Trial (DCCT) and the Epidemiology of Diabetes Interventions and Complications (EDIC) study [55–57]. Animal studies demonstrated that metabolic memory is responsible for sustained transcriptional changes in fibrotic and inflammatory genes that are involved in diabetic complications in smooth muscle cells [58, 59]. Consistent with the idea of metabolic memory, studies in humans showed that high-fat diet can induce long-lasting DNA methylation marks in proinflammatory genes that are not easily removed with diet reversal [14], although the functional significance of these changes remains to be determined.

### Physiological significance of SM programing

One possible implication of SM metabolic memory is a higher susceptibility of obesity-exposed individuals to IR and muscle loss, as a result of sustain diet-induced alterations in local gene expression. There is evidence suggesting that IR and obesity are associated with a decrease in the proportion of slow twitch fibers [60], representing the majority of soleus muscle [39], while leanness is associated with increased oxidative capacity of SM [61]. Although the present study did not directly address the effects of WSD and CR on fiber composition and metabolic properties of SM, the RNAseq analysis suggests that WSD induced the sustained downregulation of insulin signaling (*IRS1*), glycolytic (*PFKM, PFKFB1*), and mitochondrial (*UCP3*) genes and that this effect persisted after obesity was reversed with CR (Fig. 5). Interestingly, the slow twitch-specific myosin heavy chain isoform *MYH1* was also downregulated after CR (Fig. 2d). It is possible that sustained transcriptional changes observed in the present report correlate metabolic dysfunction, although this hypothesis needs further verification using functional studies.

### Transcriptional response of immunological genes

SM and systemic inflammation may play a role in the development of long-term obesity complications. Our RNAseq analysis showed that WSD induced the significant upregulation of mRNA encoding *CCL2*, which persisted after the animals were switched to CR. The upregulation of *CCL2* and a concomitant increase in proinflammatory macrophages has been recently reported in quadriceps of mice fed high-fat diet for 1 week [62]. Furthermore, both *CD163* and *TLR4* were also upregulated following CR. *CD163* is exclusively expressed in monocytes and macrophages [63] to regulate the clearance and endocytosis of hemoglobin and haptoglobin complexes by macrophages, and may thereby protect tissues from free hemoglobin-mediated oxidative damage [64]. These results are in line with previous studies that reported activation of inflammatory pathways in SM following obesity and high-fat diet [65]. As described in this manuscript, an increase in macrophage markers was observed in SM of obese nondiabetic patients [66] and in SM of mice fed high-fat diet for 3 weeks [67]. The development of obesity involves the recruitment of inflammatory CD11C+ macrophages to SM [62]. Interestingly, exposure to palmitate induces a release of IL-6 and CCL2 from macrophages [66], while conditional media from palmitate-treated macrophages sufficed to induce IR in cultured myotubes [68, 69].

### Changes in circulating cytokines

**TNFα:** We did not observe a systemic proinflammatory response and serum cytokines TNFα and IL-1 were not significantly affected by diet (Additional file 1: Figure S1). This does not rule out the possibility that local myokines production by adipose tissue and leukocytes is increased following overnutrition. Earlier studies reported that high-fat diet increases [70] and CR reduces TNFα expression in SM [71], suggesting that CR may circumvent the apoptotic effect of TNFα in SM [72]. Acute early life exposures to TNFα renders muscle cells more susceptible to impaired regeneration when inflammation is encountered in later proliferative life [73], which supports the role TNFα in muscle degeneration. Interestingly, the *TNFRSF12A* gene was significantly upregulated by WSD and downregulated by CR (Additional file 2: Table S1 and Table 2), while the transcriptional activation of this gene has been previously linked to diffuse muscle atrophy [74].

**IL-6:** In contrast to TNFα, circulating IL-6 levels were increased significantly following CR. Previous studies implicated IL-6 in mediating a proinflammatory response in patients with cancer cachexia [75, 76], in women with anorexia nervosa [77] and also in association with aging [78], sarcopenia, and muscle degeneration [79]. Furthermore, we have recently observed that IL-6 levels are significantly elevated in NHPs undergoing androgen deprivation, which was associated with a significant loss of lean mass [35]. These examples outline the involvement of IL-6 in catabolic processes associated with body wasting and cell death. There is evidence that IL-6 secretion from SM is increased after exercise [80, 81]. Furthermore, IL-6 may play a beneficial role as a regulator of glucose homeostasis during exercise. For example, the injection of recombinant IL-6 during exercise results in increased glucose infusion and glucose production rates in healthy men [82]. Additionally, IL-6 directly stimulates glucose uptake and fatty acid oxidation in myotubes in vitro [83] and improves glucose tolerance in rodent models (reviewed in [84]. IL-6 is also an important myogenic factor regulating satellite-mediated muscle hypertrophy in response to exercise [85, 86].

**MIF** and **PAI-1:** Circulating MIF and PAI-1 levels were elevated during WSD and CR, although the significance of these changes remains unclear. MIF has been shown to promote fibroblast survival and collagen synthesis in SM [87–89], while PAI-1 levels are increased after long-term glucocorticoid use, which contributed to muscle wasting and IR in mice [90] and is thought to be responsible for SM fibrosis [91].

### Limitations

The dietary effects on SM transcription need further verification using proteomic, immunological and morphological studies. General anesthesia may influence gene expression in SM, although the relative diet-specific effects are statistically significant. Due to a low sample number, the study is considered to be preliminary and need verification in a larger cohort of animals and in both sexes. For technical reason, the Luminex analysis of circulating cytokines was conducted after 4 months on a WSD and thus are not directly comparable with the RNAseq data.

## Conclusions

WSD induces reprograming of SM transcriptome, with the increased expression of profibrotic and proinflammatory genes and decreased expression of metabolic genes, which persists even after obesity is reversed by CR. This type of programing (metabolic memory) may represent an adaptive mechanism that controls metabolic and structural remodeling of SM, but may also contribute to long-term glucose intolerance and treatment resistance in obese and diabetic patients.

## Additional files

Additional file 1: Figure S1. Luminex analysis of circulating cytokines. Plasma samples were collected while on chow, 4 months on WSD and 4 months after CR, and analyzed in duplicates using Invitrogen Monkey Magnetic 29-Plex Panel, as described in “Materials and Methods”. Error bars represent SEM. Statistical significance was determined by repeated-measure one-way ANOVA, * *p* < 0.05. ** *p* < 0.01, ***p < 0.001. Abbreviations: FGF, fibroblast growth factor; IL, interleukin; G-CSF, granulocyte-colony stimulating factor; HGF, hepatocyte growth factor; VEGF, vascular endothelial growth factor; INFg, interferon gamma; MDC, macrophage-derived chemokine; I-TAC*,* interferon-inducible T cell alpha chemokine; RANTES, regulated on activation, normal T cell expressed and secreted; Eotaxin, CCL11; MIF analyte, migration inhibitor factor; TNF-a, tumor necrosis growth factor-alpha; MIP-1a, macrophage inflammatory protein-1alpha; GM-CSF, granulocyte-macrophage colony-stimulating factor; MIP-1b, macrophage inflammatory protein-1beta; IP-10, interferon-gamma-inducible protein 10; MIG, monokine induced by gamma interferon; MCP-1, monocyte chemotactic protein 1; EGF, epidermal growth factor. (PDF 998 kb)

Additional file 2: Table S1. The list of differentially expressed WSD/CR genes identified in the present study. **Table S2.** The list of differentially expressed CR/WSD genes identified in the present study. **Table S3.** Erythroid genes identified in the present study. **Table S4.** qRT-PCR primers used in the study. (XLSX 327 kb)

## Abbreviations

CR: Caloric restriction
ECM: Extracellular matrix
FFA: Free fatty acid
IR: Insulin resistance
NHP: Nonhuman primate
SM: Skeletal muscle
WSD: Western-style diet
