# Erratum to: Long-lasting effect of obesity on skeletal muscle transcriptome

**DOI:** 10.1186/s12864-017-3859-3

**Published:** 2017-06-22

**Authors:** Ilhem Messaoudi, Mithila Handu, Maham Rais, Suhas Sureshchandra, Byung S. Park, Suzanne S. Fei, Hollis Wright, Ashley E. White, Ruhee Jain, Judy L. Cameron, Kerri M. Winters-Stone, Oleg Varlamov

**Affiliations:** 1 School of Biological Sciences, University of California, Irvine, Irvine, CA 92697 USA; 2 Division of Cardiometabolic Health, Oregon National Primate Research Center, L584 505 NW 185th Ave, Beaverton, OR 97006 USA; 3 Division of Biomedical Sciences, School of Medicine, University of California, Riverside, Riverside, CA 92521 USA; 4 Department of Public Health and Preventive Medicine, Oregon Health and Science University, Portland, OR 97239 USA; 5 Division of Neuroscience, Oregon National Primate Research Center, Beaverton, OR 97006 USA; 6 Department of Neuroscience and Psychiatry, University of Pittsburgh, Pittsburgh, PA 15260 USA; 7 Department of School of Nursing, Oregon Health and Science University, Portland, OR 97239 USA

## Erratum

After the publication of this article [1] the authors found that the following errors regarding Additional file 2 had occurred:

1. The title for **Additional file 2: Table S1** should indicate the list of differentially expressed WSD/Chow genes (instead of WSD/CR).
  The correct title on the figure and within the article should therefore be:
**Additional file 2: Table S1.** The list of differentially expressed **WSD/CHOW** genes identified in the present study.
2. The title for **Additional file 2: Table S2** should indicate the list of differentially expressed CR/Chow genes (instead of CR/WSD).
  The correct title on the figure and within the article should therefore be:
**Additional file 2: Table S2.** The list of differentially expressed **CR/CHOW** genes identified in the present study.

The original article has been corrected.
