# Supplementary figures and images for: Long-lasting effect of obesity on skeletal muscle transcriptome
Source: BMC Genomics. 2017 May 25;18:411. doi: 10.1186/s12864-017-3799-y (PMC5445270; doi:10.1186/s12864-017-3799-y)

Additional file 1: Figure S1

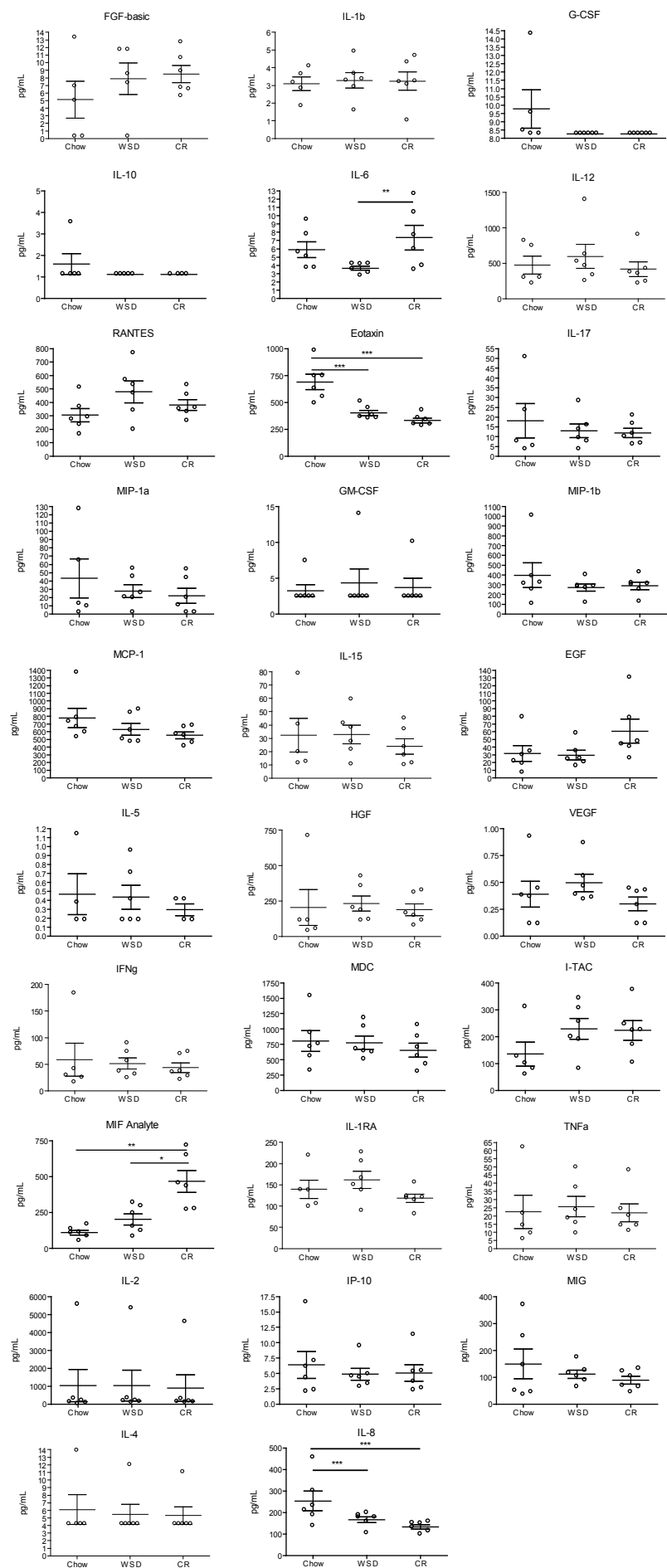

Supplement: Supplementary file 1 — Luminex analysis of circulating cytokines. Plasma samples were collected while on chow, 4 months on WSD and 4 months after CR, and analyzed in duplicates using Invitrogen Monkey Magnetic 29-Plex Panel, as described in “Materials and Methods”. Error bars represent SEM. Statistical significance was determined by repeated-measure one-way ANOVA, * p < 0.05. ** p < 0.01, ***p < 0.001. Abbreviations: FGF, fibroblast growth factor; IL, interleukin; G-CSF, granulocyte-colony stimulating factor; HGF, hepatocyte growth factor; VEGF, vascular endothelial growth factor; INFg, interferon gamma; MDC, macrophage-derived chemokine; I-TAC, interferon-inducible T cell alpha chemokine; RANTES, regulated on activation, normal T cell expressed and secreted; Eotaxin, CCL11; MIF analyte, migration inhibitor factor; TNF-a, tumor necrosis growth factor-alpha; MIP-1a, macrophage inflammatory protein-1alpha; GM-CSF, granulocyte-macrophage colony-stimulating factor; MIP-1b, macrophage inflammatory protein-1beta; IP-10, interferon-gamma-inducible protein 10; MIG, monokine induced by gamma interferon; MCP-1, monocyte chemotactic protein 1; EGF, epidermal growth factor. (PDF 998 kb) [file 12864_2017_3799_MOESM1_ESM.pdf]
